# Prognostic role of regenerating gene-I in patients with stage-IV head and neck squamous cell carcinoma

**DOI:** 10.1186/s13000-016-0526-y

**Published:** 2016-08-18

**Authors:** Mohamed Aboshanif, Yohei Kawasaki, Yasufumi Omori, Shinsuke Suzuki, Kohei Honda, Satoru Motoyama, Kazuo Ishikawa

**Affiliations:** 1 Department of Otorhinolaryngology, Head and Neck Surgery, Akita Graduate School of Medicine, Akita, Japan; 2 Department of Molecular Pathology and Tumor Pathology, Akita Graduate School of Medicine, Akita, Japan; 3 Department of Comprehensive Cancer Control, Akita Graduate School of Medicine, Akita, Japan; 4 Department of Otolaryngology Head and Neck Surgery, Akita University, Graduate School of Medicine, 1-1-1, Hondo, Akita, 010-8543 Japan

**Keywords:** REG-I, Head and neck cancer, Squamous cell carcinoma, Stage IV, Prognostic role

## Abstract

**Background:**

Regenerating gene (REG) family is composed of antiapoptotic factors and growth factors that affect epithelial cells within the digestive system. Regenerating gene-I has been studied in different cancers. However, it has never been studied in head and neck cancer. We investigated the expression of REG-I in head and neck SCC and its relevance to patient survival rates.

**Methods:**

Untreated biopsy specimens of 60 patients with stage IV head and neck SCC were collected, and the expression of REG-I was evaluated using immunohistochemistry. The association between REG-I expression and clinico-pathological features or survival status of the patients was assessed by Chi-square test, Fisher’s exact test and Kaplan-Meier method. Cox proportional hazard model was used to identify the independent prognostic factors.

**Results:**

Incidence of lymphatic permeation, vascular invasion and pathological lymph nodes was significantly higher in REG-I negative group (*p* = 0.008, 0.030 and 0.015, respectively). Overall and cancer-free survival rates were significantly higher in REG-I positive group (*p* = 0.000434 and 1.0847E-8, respectively). Univariate analysis showed that REG-I was an independent prognostic factor for predicting long-term overall survival (*p* = 0.002), and multivariate analysis showed that REG-I and lymphatic permeation were independent prognostic factors for predicting long-term disease-free survival (*p* = 0.001 and 0.022, respectively).

**Conclusion:**

Our results showed for the first time that, REG-I is expressed in head and neck SCC. REG-I expression is associated with a longer survival status. We conclude that, REG-I might be a prognostic marker in head and neck SSC and should be further investigated.

## Background

Head and neck cancers include malignant neoplasms that arise from many sites within the upper aerodigestive tract, with the most common sites being the oropharynx, hypopharynx, larynx, and oral cavity [1]. Most of these epithelial malignancies are squamous cell carcinoma of the head and neck (SCCHN), for which the most important risk factors are tobacco and alcohol consumption [2]. However, there is increasing evidence that the human papilloma virus (HPV) has a role [3]. About two-thirds of patients with SCCHN present with advanced stage disease, mainly involving the regional lymph nodes; and 10 % of patients have distant metastasis at initial presentation [4]. Detection of factors that affect the prognosis of these advanced cancers is important to obtain an even better outcome. Regenerating gene (REG) was firstly isolated as up-regulated gene in regenerating islet cells [5]. Regenerating gene family members that have been reported in humans include REG Iα, REG Iβ, REG III, HIP/PAP and REG IV [6], with an association between REG-I expression and islet cell replication [7]. It has recently been shown that REG-I expression predicts long-term survival in locally advanced thoracic squamous cell esophageal cancer [8]. However, to date, there have been no reports regarding the expression of REG-I in head and neck squamous cell carcinoma. In this study, we investigated REG-I expression and its correlation with the clinico-pathological features and survival status in stage IV head and neck squamous cell carcinoma.

## Methods

### Patients and tissue samples

The medical records of 60 patients who were treated for stage IV head and neck squamous cell carcinoma at Akita university hospital were investigated. Of these patients, 22 (36.7 %) were diagnosed as T1 or T2, and the other 38 (63.3 %) were diagnosed as T3 or T4. The age of the patients ranged from 28 to 85 years, with a mean age of 63.7 ± 11SD. All patients had received preoperative radiotherapy of 40 Gray and chemotherapy (taxotel or docetaxel 10 mg/m^2^/week) followed by surgery. The clinico-pathological characteristics of the patients are shown in Table 1.

**Table 1:** Patients’ characteristics

Variables	Number of cases (*N* = 60)
Age (≤65 />65)	36/24
Sex (Male/Female)	50/10
T-classification (T1-T2/T3-T4)	22/38
N-classification (N0/N1-3)	3/57
Lymphatic permeation (Yes/No)	32/28
Vascular invasion (Yes/No)	22/38
Pathological lymph nodes (Yes/No)	25/35
REG-I expression (Positive/Negative)	36/24
Grades (I, П, Ш)	19/22/19
Site (Oropharynx, Hypopharynx, Tongue, Larynx)	13/19/15,13
Events	
Death (overall survival) Death or recurrence (disease free survival)	14 40

The expression of REG-I in biopsy specimens, obtained from all patients prior to therapy, was examined to avoid the effect of radio-chemotherapy on the results.

### Immunohistochemistry

We prepared deparaffinized sections of untreated biopsy specimens of head and neck cancer for immunohistochemical staining for REG-I; they were initially autoclaved for 15 min at 121 °C, then were blocked with 0.3 % hydrogen peroxide in methanol for 30 min at room temperature and with 10 % BSA/TBS for 30 min at room temperature. All sections were kept overnight at 4 °C in phosphate-buffered saline containing anti-REG-I monoclonal antibodies (1:400 dilution, 2.5 μg/mL; BioVendor Laboratory Medicine, Inc., Evropska, Czech Republic), and were subsequently incubated for 20 min with Envision (Dako Corporation, Copenhagen, Denmark). The signal was detected by incubating the sections with diaminobenzidine solution (Dako) and hydrogen peroxide for one minute. We used image-J software as an objective method to measure the intensity of immunohistochemical staining for REG-I in biopsy specimens of head and neck cancer.

### Statistical analysis

It was performed using the IBM SPSS version 20 software for Windows. Chi-square test and Fisher’s exact test were used to check the association of categorical variables. The Kaplan–Meier method was used to estimate survival rate, and the log-rank test was used to analyze survival differences. Overall survival time was determined as the time from tumor diagnosis to death from any cause. Disease free survival time was defined as the time from treatment to tumor recurrence or death.

Univariate and multivariate analyses were performed using a Cox proportional hazards model to identify independent prognostic factors. A statistically significant difference was considered with probability values of <0.05.

## Results

### REG-I expression was found to be either positive or negative according to REG-I index

**Fig. 1:**
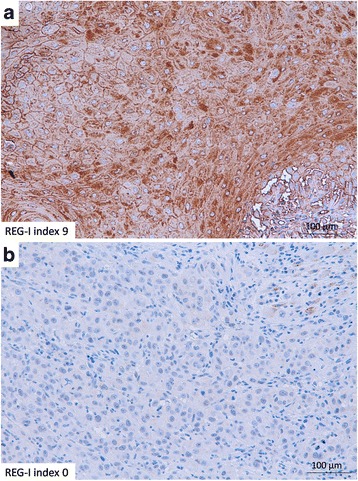
Immunohistochemical analysis of REG-I in biopsy specimens. Representative immunohistochemical analysis of REG-I expression in (**a**) a REG-I positive specimen with a REG-I index of 9, and (**b**) a REG-I negative specimen with a REG-I index of 0 (Scale bar: 100 μm)

We found that REG-I staining of all specimens fell into one of three groups; weak, moderate or strong staining, which we scored as 1–3, respectively. We further measured the area of REG-I staining. Staining was scored as 0, 1, 2 or 3 if less than 10 %, 10 % or more but less than 50 %, 50 % or more but less than 90 %, or 90 % or more of the tumor cells were stained, respectively. Finally, we calculated a REG-I index (0–9) as the REG-I stained area score (0–3) × REG-I intensity score (1–3) (Fig. 1). This method was based on a previous report on esophageal cancer [8].

After analysis of all specimens and calculation of the REG-I index, we found that the patients could be grouped into two main categories; those with a REG-I index ≥6, which showed either strong staining with ≥50 % stained area or moderate staining with >90 % stained area, where most of specimens got REG-I index 6, and the remaining specimens got REG-I index 9, and considered as REG-I positive. The other category includes those with a REG-I index ˂6, which showed either strong staining with <50 % stained area, moderate staining with <90 % stained area, or weak staining, where most of specimens got REG-I index 4 and the remaining specimens got REG-I index 3, 2 and 0, and considered as REG-I negative (Table 2). After dividing the specimens into two categories, we tried to test if there is a difference between both groups in the clinico-pathological parameters and survival rates.

**Table 2:** REG-I Index shows that all patients fall into two groups; one group with index ≥6 and the other group with index <6

REG-I index	Number of cases	Total
9	2	36 positive cases
6	34	
4	17	24 Negative cases
3	3	
2	3	
0	1	

### Association of REG-I expression to the survival rates

We used Chi-square test and Fisher’s exact test to compare the clinicopathological parameters between the REG-I positive and negative groups (Table 3). This analysis indicated that the incidence of lymphatic permeation, vascular invasion and pathological lymph nodes was significantly higher in the REG-I negative group than in the REG-I positive group (*p* = 0.008, 0.030 and 0.015, respectively) (Table 3).

**Table 3:** Results of Fisher’s exact test and Chi-squared test in comparison between REG-I positive and negative groups

REG-I staining Variables	REG-I positive (*N* = 36)	REG-I negative (*N* = 24)	*P* value
Age			
≤ 65 > 65	21 15	15 9	0.793
Sex			
Male Female	30 6	20 4	1.000
Lymphatic permeation			
Yes No	14 22	18 6	0.008
Vascular invasion			
Yes No	9 27	13 11	0.030
T-classification			
T1/T2 T3/T4	15 21	7 17	0.416
N-classification (pretreatment)			
Yes No	35 1	22 2	0.558
Pathological lymph nodes			
Yes No	10 26	15 9	0.015
Grade			
Grade I Grade П Grade Ш	12 11 13	7 11 6	0.459^a^
Site of primary tumor			
Oropharynx Hypopharynx Tongue Larynx	8	5	0.944^a^

^a^Chi-squared test

**Fig. 2:**
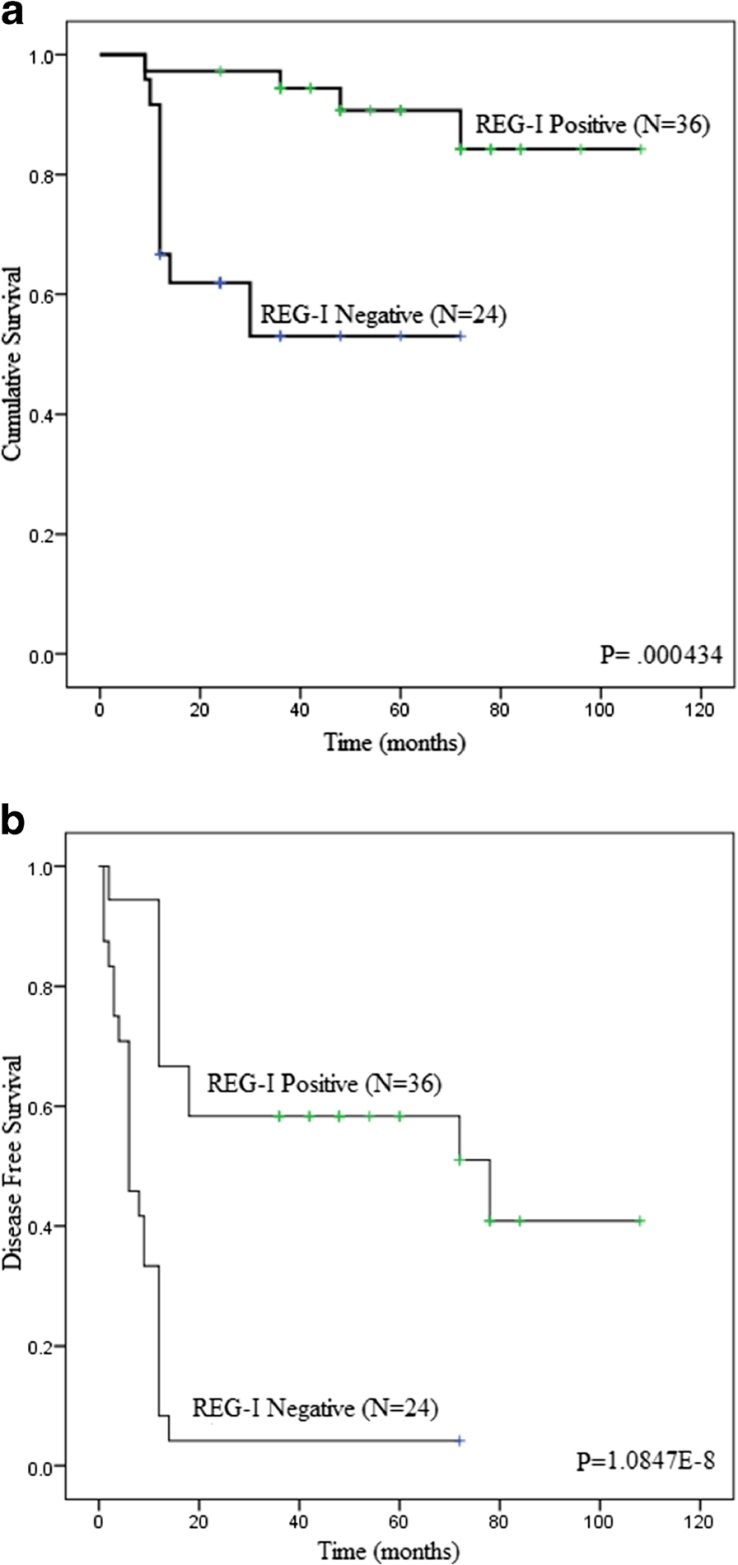
Association of REG-I expression with patient survival rates. Overall (**a**) and cancer free (**b**) survival rates of REG-I positive and REG-I negative head and neck cancer patients analyzed using Kaplan Meier curves

Figure 2 shows Kaplan–Meier survival curves of REG-I positive and negative groups. The overall survival rate was significantly higher in the REG-I positive group than in the REG-I negative group (5-year survival rates were 86 and 52 %, respectively; *p* = 0.000434). Also, the cancer-free survival rate was significantly higher among patients with a positive REG-I index than among those with a negative index (5-year survival rates were 59 and 8 %, respectively, *p* = 1.0847E-8).

Table 4 shows the results of univariate analysis. We included 10 covariates: age, sex, lymphatic permeation (yes / no), vascular invasion (yes / no), T-classification (T1,2 / T3,4), N-classification (yes/no), presence of postoperative positive lymph nodes (yes / no), tumor grade (low, intermediate, high), site of tumor (hypopharyngeal, oropharyngeal, tongue, larynx and REG-I expression (positive / negative).

**Table 4:** Univariate analysis of overall and disease-free survival rates

Survival rates Variables	Overall survival		Disease free survival	
	*P* value	95 % CI (HR)	*P* value	95 % CI(HR)
Age ≤ 65/>65	0.491	0.506–4.124 (1.445)	0.606	0.445–1.604 (0.845)
Sex Male/Female	0.318	0.046–2.711 (0.355)	0.818	0.505–2.378 (1.096)
Lymphatic permeation Yes/No	0.584	0.465–3.896 (1.346)	0.000088	2.161–10.087 (4.669)
Vascular invasion Yes/No	0.193	0.702–5.769 (2.012)	0.004	1.352–4.805 (2.548)
T-Classification T1-T2/ T3-T4	0.653	0.423–3.943 (1.292)	0.060	0.972–4.279 (2.039)
N-Classification (pretreatment) Yes/No	0.082	0.057–1.184 (0.261)	0.060	0.096–1.047 (0.317)
Pathological lymph nodes Yes/No	0.131	0.783–6.591 (2.272)	0.003	1.385–5.036 (1.385)
Grade Grade I/ Grade П/ Grade Ш	0.459	0.404–1.505 (0.780)	0.797	0.648–1.395 (0.951)
Site (Oropharynx, Hypo-pharynx, Tongue, Larynx)	0.721	0.640–1.904 (1.104)	0.426	0.830–1.556 (1.136)
REG-I expression Positive/Negative	0.002	0.042–0.497 (0.144)	0.000002	0.098–0.379 (0.193)

Univariate analysis showed that REG-I expression (*p* = 0.002) was significant prognostic factor affecting the overall survival with hazard ratio (95 % confidence interval) of 0.144 (0.042– 0.497), while REG-I expression (*p* = 0.000002), lymphatic permeation (*p* = 0.000088), vascular invasion (*p* = 0.004), and the presence of pathological lymph nodes (*p* = 0.003) were significant prognostic factors affecting the disease-free survival.

Multivariate analysis using the Cox proportional hazard model showed that REG-I expression (*p* = 0.001) and lymphatic permeation (*p* = 0.022) are independent prognostic factors for prediction of long-term disease-free survival. The hazard ratio of REG-I expression (95 % confidence interval) for the disease-free survival rate was 0.298 (0.141–0.626) (Table 5).

**Table 5:** Multivariate analysis of disease-free survival rate

Survival rate Variables	Disease free survival	
	*P* value	95 % CI (HR)
Lymphatic permeation Yes/No	0.022	1.182–8.744 (3.215)
Vascular invasion Yes/No	0.790	0.422–1.929 (0.902)
Pathological lymph nodes Yes/No	0.535	0.607–2.610 (1.259)
REG-I expression Positive/Negative	0.001	0.141–0.626 (0.298)

## Discussion

There have been no previous reports of investigation of the expression of REG-I in head and neck squamous cell carcinoma. In the present study, 36 cases were positive for REG-I expression, mainly with a REG-I index of 6. We compared those patients with the REG-I negative group (24 patients) and found that the REG-I positive group had a lower incidence of lymphatic permeation (absent in 22 patients (61 %) in the positive group but only six patients (25 %) in the negative group) and vascular invasion (absent in 27 patients (75 %) in the positive group and 11 patients (46 %) in the negative group); and that pathological lymph nodes were absent in 26 patients (72 %) in the positive group, but only nine patients (38 %) in the negative group. Also, it has been reported that REG-Iα expression was significantly associated with the prevalence of vascular invasion [9, 10].

But we could not find any significant difference between the two groups in terms of age, sex, clinical T-stage, N-stage, or site of primary tumor. In lung SCC, there was no significant difference in age, sex or tumor stage between REG-Iα positive and negative groups [11].

Furthermore, as in case of esophageal squamous cell carcinoma, we could not find any difference in regards to grade of differentiation between the two groups [8]. It has been reported that, REG-I was frequently expressed by gastric carcinomas that were not well differentiated, and identifying REG-I expression may help to detect aggressive tumors [10].

Although, we could not prove that lymphatic permeation, vascular invasion or pathological lymph nodes had a significant effect on the overall survival, but we proved that they could significantly affect the disease-free survival. On the other hand, it has been reported that vascular invasion, lymph node metastasis and REG-Iα expression were significantly predictive of overall survival in gastric tumors [9, 10]. Nevertheless, we found that patients with a REG-I index of ≥6 had significantly higher overall and disease free survival rates than patients with a REG-I index of <6.

To date, only two forms of the human REG-I gene, REG-Iα and REG-Iβ, have been identified. The REG-Iα, REG-Iβ and RS (REG-related sequence) genes are found in a 95-kb region on chromosome 2p12 [12]. REG gene family comprises antiapoptotic factors and growth factors that affect islet cells, neural cells and epithelial cells of the digestive system [13, 14]. It also encodes proteins involved not only in regeneration of damaged tissues, but also in the growth of cancers [15–29].

We found that REG-I expression is associated with longer survival in advanced head and neck cancer treated by chemoradiotherapy; our results are consistent with previous results regarding esophageal cancer [8].

On the other hand, Macadam et al. found that expression of REG mRNA was associated with a poor prognosis in surgically treated colon carcinoma patients, and that the REG mRNA status was the only independent factor for tumor recurrence [30]. It has also been demonstrated that, REG-I positivity is associated with a worse overall survival rate in patients with surgically treated gastric carcinoma. In that study, REG-I expression was an independent prognostic factor for overall survival by multivariate analysis, but not for disease-free survival [10]. This could be explained by the differences between gastrointestinal cancers and esophageal cancers; most esophageal cancers are SCCs, whereas other gastrointestinal cancers are adenocarcinomas [11]. This conflicting findings regarding Reg-I positivity and its effect on prognosis could be related to treatment policy.

Finally, we have used a REG-I staining score developed by one of our co-authors in a previous report for esophageal cancer, but we have used image-J software as an objective tool in measuring the area and strength of the stain to avoid subjectivity. We also have used immunohistochemistry, in spite of its known limitations to determine the function of gene expression [31], to evaluate REG-I expression and its correlation with the survival rate. Further investigation of REG-I expression through evaluation of REG messenger RNA is recommended [10].

## Conclusion

The results of our study indicate that REG-I is expressed by advanced head and neck squamous cell carcinoma and that REG-I positivity, in cases treated with chemo-radiotherapy, is associated with a lower incidence of lymphatic permeation, vascular invasion and pathological lymph nodes. These findings may help in selecting the appropriate therapy for high-risk patients (smokers, positive family history, etc.) with REG-I positive biopsy specimens. Furthermore, REG-I is a good prognostic tool in late stage head and neck carcinoma. Further studies are necessary to determine the role of REG-Iα in head and neck cancer.

## Abbreviations

HPV, human papilloma virus; REG, regenerating gene; SCCHN, squamous cell carcinoma of the head and neck; SCCs, squamous cell carcinomas; WD, well differentiated
